# Endophytic Fungi Isolated from *Baccharis linearis* and *Echinopsis chiloensis* with Antifungal Activity against *Botrytis cinerea*

**DOI:** 10.3390/jof8020197

**Published:** 2022-02-18

**Authors:** Paulo Castro, Rodolfo Parada, Carlos Corrial, Leonora Mendoza, Milena Cotoras

**Affiliations:** Faculty of Chemistry and Biology, University of Santiago of Chile, Bernardo O’Higgins Avenue 3363, Estación Central, Santiago 9160000, Chile; paulo.castro@usach.cl (P.C.); rodolfo.paradaf@usach.cl (R.P.); carlos.corrial@usach.cl (C.C.)

**Keywords:** antifungal compounds, *Baccharis linearis*, *Botrytis cinerea*, *Echinopsis chiloensis*, endophytic fungi

## Abstract

*Botrytis cinerea* is one of the most important phytopathogens in agriculture worldwide, infecting economically important crops. The main control of this fungus is by synthetic fungicides, causing the selection of resistant isolates. Compounds produced by endophytic fungi have been shown to have antifungal activity against this pathogen and can be used as an alternative to synthetic fungicides. The aim of this work was to isolate endophytic fungi from Chilean foothills in the Metropolitan Region. Ten fungi were isolated from *Echinopsis chiloensis* and *Baccharis linearis*, however, only two isolates inhibited the mycelial growth of *B. cinerea* by antibiosis and were identified as *Epicoccum* sp. and *Pleosporales* sp. Extracts at 200 mg L^−1^ from *Epicoccum* sp. and *Pleosporales* sp. showed antifungal activity against *B. cinerea* of 54.6 and 44.6% respectively. Active compounds in the *Epicoccum* sp. extracts were mainly alkaloids and phenolic compounds; meanwhile, in the *Pleosporales* sp. extracts, terpenes and/or saponins were responsible for the antifungal activity.

## 1. Introduction

*Botrytis cinerea* is a phytopathogenic fungus that produces the disease called gray mold within a wide range of plant species [1] and it is one of the most important plant pathogen fungi globally, causing significant economic losses during storage and transportation [1]. The main control method of this disease is by synthetic compounds [1]. However, this control method affects the environment [2] and the selection of resistant isolates of the fungus [3]. In Chile, *B. cinerea* resistant isolates have also been described in ‘Thompson Seedless’ table grapes. Several fungicides were tested and only around 20% of the isolates were sensitive to all these fungicides [4]. Hence, the development of new antifungals to control *B. cinerea* requires urgent attention.

In recent years, attention has focused on the development of new strategies based on chemical compounds obtained from microorganisms for the control of *B. cinerea*. The main advantages of these compounds are that they last a short time in the environment and have a varied mechanism of action that prevents the development of resistant strains of the phytopathogen [5].

In the last decade, plant endophytic fungi have increased due to their potential as producers of active secondary metabolites [6]. Most plants are colonized by endophytic fungi; it has been reported that plants are the host of one or several strains of endophytes [7]. These are microorganisms that can live inside tissue of living plants without showing any disease symptoms [8]. Endophytes can produce different secondary metabolites with biological activities such as antibacterial, fungicidal, and algicidal properties [9,10,11]. Different compounds from endophytes possessing antifungal activity against *B. cinerea* have been described so far, including monoterpene derivatives obtained from the plant endophytic fungus *Pestalotiopsis foedan* [12], hydroxyanthraquinone derivatives from *Coniothyrium* sp. [13], Griseofulvin, from the endophyte *Xylaria* sp. and dihydrocoumarin derivatives from the fungus *Pezicula* sp. [14,15,16].

Regarding endophytic fungi with antifungal activity isolated from Chilean plants, few works have been published. Eight gymnosperm species of southern of Chile, *Araucaria araucana*, *Austrocedrus chilensis*, *Fitzroya cupressoides*, *Pilgerodendron uviferum*, *Podocarpus nubigena*, *Podocarpus saligna*, *Prumnopitys andina*, and *Saxegothaea conspicua* were analyzed and thirty-eight endophytic fungi, some of these producing antifungal compounds, were isolated [17]. Also, it has been described that foliar endophytic fungi isolated from the Chilean tree *Embothrium coccineum* present low antifungal activity against *B. cinerea* [18] and, more recently, endophytic fungi from the endemic plant *Echinopsis chiloensis* with antifungal activity against *B. cinerea* have been described by Vidal et al. [11].

Different criteria have been considered to find endophytic fungi with antifungal activity. For instance, perennial plants of endemic origin that inhabit environments with great biodiversity increase the probability of finding diverse endophytic fungi [19]. Central Andean Precordillera of Chile has a Mediterranean climate with a very high geographic and climate heterogeneity [20,21]. These ecological conditions have allowed the development of a wide range of endemic and native plant species [21]. Research of endophytic fungi of these plants is barely developed as we mentioned above; this offers an opportunity to find endophytic fungi or their metabolites with biotechnological potential [22]

Plants such as *E. chiloensis* and *B. Linearis* inhabit this place. *E. chiloensis* is the most prominent cactus in central Chile and it can live in Mediterranean pluvial–seasonal and xeric–oceanic climates [23], and only one endophytic fungus has been described on this plant [11]. On the other hand, *B. linearis* is a dioecious, small to a medium-sized evergreen shrub that lives in drylands and is found in the Coastal and Andes Mountains in Central Chile and in Argentinian Patagonia [24]. In this plant, only mycorrhizal (*Glomus* sp.) and bacterial endophytes have been found but, isolation of endophytic fungi have not been described for this plant [25].

Given the great diversity of ecological systems in the area, it is expected that these plants contain various endophytic fungi with bioactive secondary metabolites that inhibit the growth of *B. cinerea*. Consequently, the aim of this work was to find new endophytic fungi obtained from *E. chiloensis* and *Baccharis linearis*, growing in the Central Andean Precordillera of Chile, with antifungal activities against *B. cinerea*.

## 2. Materials and Methods

### 2.1. Collection of Plant Materials

Samples were obtained from plant roots (*E. chiloensis* and *B. linearis*) in Camino Real, El Ingenio in central Andean Precordillera of Chile (Latitude: −33.75002; Longitude: −70.283477). Healthy tissue from plants were recollected in April 2019. Samples were kept at 4 °C in sterile Falcon tubes until use.

### 2.2. Isolation of Endophytes

Tissue surface sterilization was carried out as described by Silva-Hughes et al. [26] with modifications. Tissues were immersed in 70% ethanol and 0.2% Tween 20 for three minutes, then washed with sterile distilled water and treated with 2.5% sodium hypochlorite for three minutes. Lastly, tissues were washed with sterile distilled water once more. Sterilized tissues were then cut into small fragments (0.5 cm), placed in Petri dishes containing potato dextrose agar (PDA), and then supplemented with kanamycin sulfate (0.05 mg L^−1^) and chloramphenicol (0.034 mg L^−1^). Plates were incubated for fourteen days at 22 °C. Fungi obtained were transferred to new PDA plates and incubated at 22 °C. This procedure was repeated several times until obtaining a pure culture. The isolated endophytes from *E. chiloensis* and *B. linearis* were designated Ech1 to Ech6 and Bl1 to Bl4, respectively.

### 2.3. Antifungal Assays against B. cinerea

The strain G29 of *B. cinerea* was used in this study. This strain was isolated from infected grapes (*Vitis vinifera*) [27].

#### Confrontation Assays

The evaluation of the antifungal activity of the endophytes against *B. cinerea* was carried out using dual confrontation assays as described by Chen et al. [28], with modifications. Small discs of PDA culture medium with mycelium of the isolated endophytes and *B. cinerea* were inoculated in opposites sides of PDA plates (6 cm apart) supplemented with antibiotics. *B. cinerea*, inoculated in one side of the PDA Petri dishes, was used as a control. Inhibition percentage was calculated using the radial mycelial growth of *B. cinerea*, confronting the endophyte (Ri) and the radial mycelial growth of *B. cinerea* in the control (Rc) according to the formula (Rc − Ri)/Rc × 100. These experiments were done in triplicate.

### 2.4. Identification of Endophytes

For the molecular identification of the endophytic fungi, genomic DNA was purified using the CTAB method [29]. Around 200 mg of endophyte tissue obtained from axenic cultures was placed in a Falcon tube with 800 µL of CTAB buffer (3% CTAB, 1.4 M NaCl, 20 mM EDTA and 100 mM Tris-HCl pH 8.0). The tissue was vortexed using 4 mm diameter glass spheres for 3 min. Disrupted tissue was incubated at 60 °C for 30 min. Then, it was centrifuged at 8400× *g* for 10 min at room temperature, and the supernatant was separated from the cellular debris. The recovered supernatant was treated with 2 ng µL^−1^ RNAse at 37 °C for 30 min. Next, 800 µL of chloroform:isoamyl alcohol (24:1) was added, and the aqueous phase was recovered after centrifugation for 14,196× *g* for 10 min at room temperature. Later, 800 µL of cold isopropanol was added and incubated at 20 °C for 2 h. Subsequently, the solution was centrifuged for 14,196× *g* for 10 min at 4 °C. After, 500 µL absolute ethanol was added to the pellet and dried using a paper towel. Finally, DNA was resuspended in DNAse free water.

ITS regions of the rDNA and β-tubulin gene were analyzed to identify the endophytic fungi [30]. To amplify the ITS sequences, the forward primer ITS-1 (5′-TCCGTAGGTGAACCTGCGG) and reverse primer ITS-4 (5′-TCCTCCGCTTATTGATATGC) were used [31], while to amplify β-tubulin marker, the forward primer BT2aF (GGTAACCAAATCGGTGCTGCTTTC) and reverse primer BT2bR (ACCCTCAGTGTAGTGACCCTTGGC) were used [32].

PCR reaction was performed in 50 µL volumes, containing 2 µL of genomic DNA, 1 µL of forward primer (10 µM), 1 µL of reverse primer (10 µM), 21 µL of nuclease-free water, and 25 µL of GoTaq^®^ Green Master Mix 2× (Promega, Madison WI, USA). PCR reaction mixtures for amplify ITS region were subjected to an initial denaturation at 94 °C for 3 min, and 38 cycles using the following temperatures: 94 °C for 40 s, 55 °C for 45 s, and 72 °C for 40 s; finally, an elongation was carried out at 72 °C for 5 min. The amplification program for β-tubulin PCR began with an initial denaturation at 94 °C for 5 min and 35 cycles using the following temperatures: 94 °C for 45 s, 55 °C for 45 s, and 72 °C for 2 min; finally, an elongation was carried out at 72 °C for 10 min. PCR reactions were performed in an Eppendorf^®^ (Hamburg, Germany) MasterCycler Personal. Finally, the PCR products were sequenced by the Genomic and Bioinformatic Center of the Universidad Católica de Chile using automatic sequencing ABI PRISM 3100.

Electropherograms were manually edited using Geneious Prime^®^ (Biomatters Ltd., Auckland, New Zealand) 2021.01 version software. A consensus sequence was created using both ITS and β-tubulin sequences, BLASTn tool was used to look for similarities with other fungi. Finally, using Geneious Prime software, consensus sequences tree construction was carried out using sequences obtained using ClustalW algorithm. Phylogenetic trees were constructed using the Tamura-Nei genetic distance model using the neighbor-joining method. Resampling for each dendrogram was 1000 using Geneious Prime software.

### 2.5. Extraction of Secondary Metabolites from the Most Active Endophytic Fungi

For the extraction of secondary metabolites, fungi were inoculated in PDA plates on a cellophane layer and incubated for seven days. After this, cellophane containing the fungi was discarded. Remaining culture medium was extracted using ethyl acetate. The organic phase was evaporated using a rotary evaporator at 40 °C. PDA without endophyte was used as negative control.

### 2.6. Antifungal Activity against B. Cinerea of the Extracts

The antifungal activity of the extracts was evaluated in vitro. Extracts dissolved in acetone were added to PDA plates at 50, 100, and 200 mg L^−1^. Acetone was added to PDA plates as a negative control. The final acetone concentration was identical in the control and treatment assay. After acetone evaporation in a laminar flow cabinet, the culture media were inoculated with 0.5 cm agar disks from an active growing culture of *B. cinerea*. Cultures were incubated in the dark at 22 °C for three days. Mycelium diameter was measured daily in two perpendicular directions. Inhibition percentages were calculated after 72 h of incubation. These experiments were done in triplicate.

A bioautography was carried out using thin-layer chromatography (TLC) to identify what fraction of the extract containing the diffusible compounds had antifungal activity [33,34]. Extract dissolved in methanol was placed in a TLC plate (silica gel 60 F254, Merck, Santiago, Chile) and separated with methanol:chloroform (9:1) as an eluent system. The separation of the compound in the TLC was visualized using UV light at 254 nm. Mobile phase methanol:chloroform (9:1) was placed in TLC plate as solvent control. Bioautography using the TLC with the separated compounds was carried out as described by Vidal et al. [11].

A preliminary characterization of the antifungal compounds in the extracts from the isolates Ech4 and Bl1 was performed using different stain solutions. Extracts were separated in TLC plates using chloroform:methanol (9:1) as an eluent system and stained with the following solutions: sulfuric acid (25% *v*/*v*) for organic compounds, Dragendorff’s reagent spray solution (Merck) for alkaloids, solution of iron (III) chloride (2% *w*/*v* iron chloride, methanol 50% *v*/*v* and 50% *v*/*v* water) for phenolics compounds, a solution of vanillin–sulfuric acid (3.5% *w*/*v* vanillin in methanol and 0.625% *v*/*v* of sulfuric acid) for terpenoids [35] and saponins [36].

### 2.7. Statistical Analysis

For all the non-parametrical statistical analyses, GraphPad Prism 6.01 was used. Statistical significance between treated groups and the control group by multiple t-tests and Holm-Sidak method are indicated with asterisks (*p* < 0.05).

## 3. Results

### 3.1. Isolation of Endophytes

**Figure 1:**
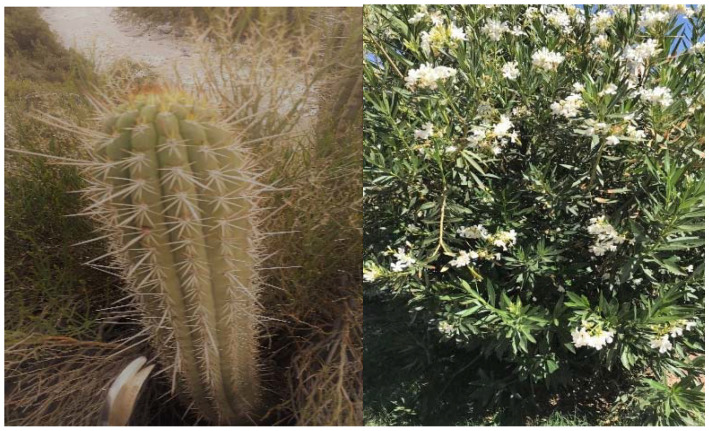
Recollected plants from the Andean Precordillera of Chile. *E. chiloensis* (**left**) and *B. linearis* (**right**).

Endophytic fungi were isolated from root of the endemic plant *Echinopsis chiloensis* and the native plant *Baccharis linearis* (Figure 1).

Six endophytic fungi were isolated from apparently healthy *E. chiloensis* and four fungi from *B. linearis* (Table 1).

**Figure 2:**
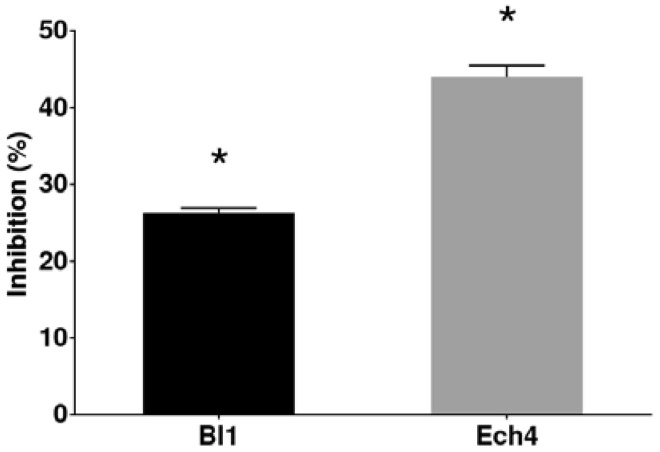
Antifungal effect of isolate Bl1 (black bar) and isolate Ech4 (grey bar) on *B. cinerea* growth. Each bar represents the average inhibition percentage of three experiments ± SD. Statistical significance when comparing treated groups and control group by multiple t-tests and Holm-Sidak method are indicated with asterisks (*p* < 0.05).

Evaluation of the antifungal activity against *B. cinerea* of the isolated fungi was carried out by using the dual confrontation assay. Among these, only two isolates, Bl1 and Ech4, showed a significative inhibition of the mycelial growth of *B. cinerea* (*p* < 0.05). The calculated inhibition values were 26.4 and 44.0% for Bl1 and Ech4, respectively (Figure 2).

**Figure 3:**
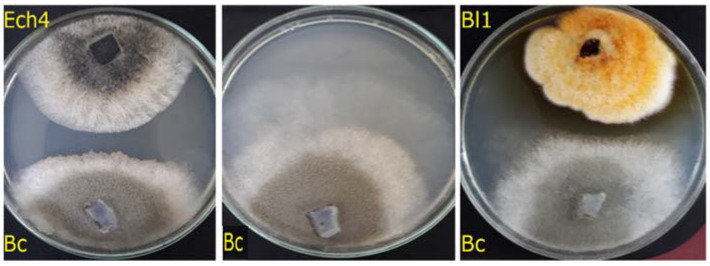
Effect of the isolates Ech4 (**upper left**) negative control (**center**) and Bl1 (**upper right**) on the mycelial growth of *B. cinerea* (**bottom** of both pictures).

The formation of an inhibition halo between the mycelia of both isolates, Ech4 and Bl1, and *B. cinerea* (Figure 3) suggests that the antifungal effect was produced by diffusible compounds (antibiosis) secreted by Ech4 and Bl4.

### 3.2. Antifungal Activity against B. cinerea of the Extracts

**Figure 4:**
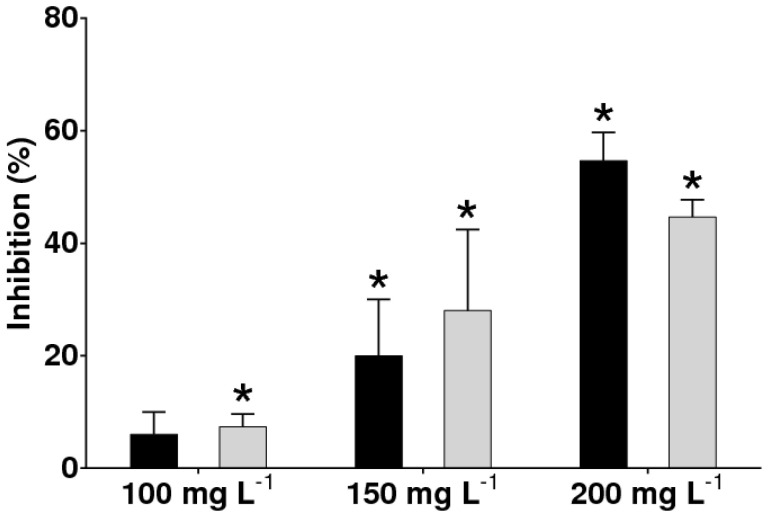
Antifungal activity of the extracts obtained from the endophytic fungi Bl1 (black bars) and Ech4 (grey bars) against *B. cinerea*. Each bar represents the average inhibition percentage of *B. cinerea* cultured in three experiments per treatment ± SD. Statistical significance when comparing treated groups and control group by multiple *t*-tests and Holm-Sidak method are indicated with asterisks (*p* < 0.05).

Extracts of the secondary metabolites produced from Ech4 and Bl1 were obtained, and the antifungal activity of these extracts was evaluated against *B. cinerea* (Figure 4). The extract obtained from the endophytic fungus Bl1 showed a higher antifungal activity against *B. cinerea* than the extract obtained from the endophytic fungus Ech4 at the tested concentrations. The extracts from Bl1 and Ech4 showed a higher antifungal activity at 200 mg L^−1^, being 54.6 and 44.6%, respectively.

**Figure 5:**
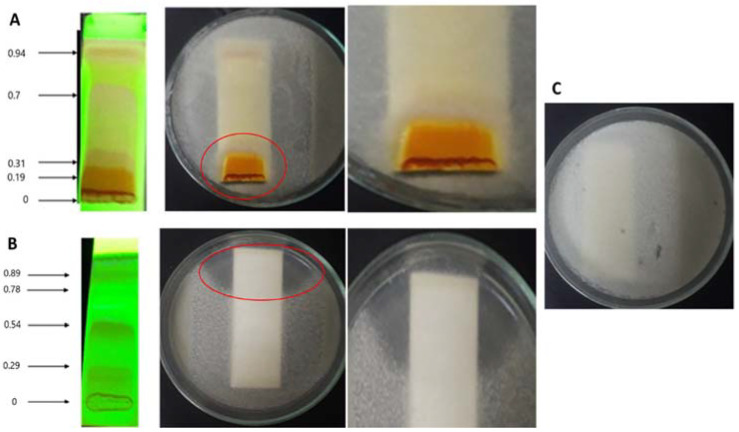
Bioautography assay for the extracts. (**A**) Chromatogram of the extract obtained from Bl1 visualized using UV light (254 nm) (mobile phase CHCl_3_:MeOH 9:1) (**left**). Bioautography of the extract Bl1 (**center**). Zone of inhibition (**right**). (**B**) Chromatogram of the extract obtained from Ech4 visualized using UV light (254 nm) (**left**) (mobile phase CHCl_3_:MeOH 9:1). Bioautography of the extract Ech4 (**center**). Zone of inhibition (**right**) Red circle indicates zone of inhibition. (**C**) Solvent control (mobile phase CHCl_3_:MeOH 9:1).

Additionally, to identify what fraction of the extracts was responsible for the antifungal activity, a bioautography assay was carried out using 2 mg of both extracts (Figure 5). Results showed that not all the compounds in the extracts had antifungal activity. Extract of the isolate Bl1 showed an inhibition halo for compounds with lower Rf values; meanwhile, the extract obtained from Ech4 also showed an inhibition halo in the zone of the compounds with higher Rf values (Figure 5).

For the preliminary characterization of the active compounds, different stain solutions were used. The bioactive fraction of the Bl1 extract was positive for Dragendorff’s reagent and iron chloride (III) solution, therefore this fraction would contain compounds of the alkaloid family, with the presence of phenolic groups, while the bioactive fraction of extract from Ech4 was positive for the vanillin/sulfuric acid solution, indicating that it could contain terpenes and saponins (Results not shown).

### 3.3. Identification of Endophytes with Antifungal Activity

**Figure 6:**
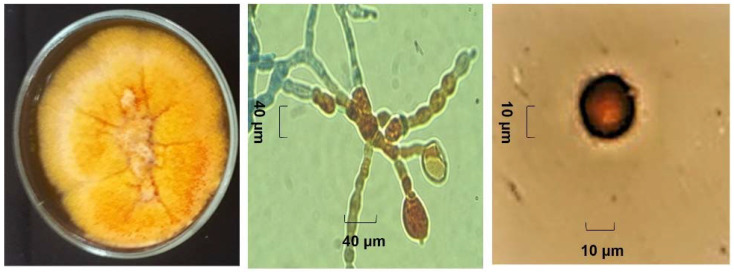
Morphological examination of the isolate Bl1. Colony on PDA (**Left**), sporodochia (**center**), and conidium (**right**).

**Figure 7:**
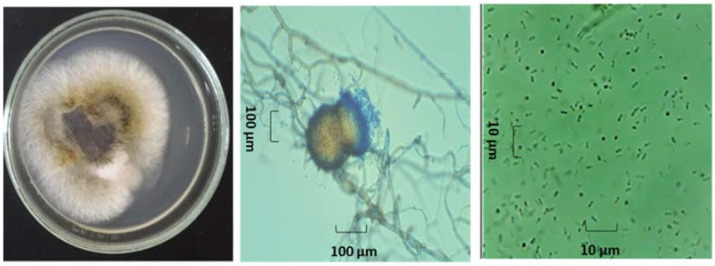
Morphological examination of the isolate Ech4. Colony on PDA (**left**), hyphae and conidiomata picnidial (**center**), and conidia (**right**).

For the morphological identification, axenic cultures of both endophytes (Ech4 and Bl1) were observed with or without a lactophenol cotton blue stain under a light microscope. The isolate Bl1 developed orange-pigmented, septate vegetative hyphae with branched growth. A single globular orange conidia was developed in each sporodochium. (Figure 6). On the other hand, the isolate Ech4 showed in PDA medium a branched hyaline septate hypha, yellow-pigmented conidiomata pycnidial globose, covered with some hyphal outgrowths and ovoid shaped small conidia smooth- and thin-walled, hyaline, aseptate. (Figure 7).

**Figure 8:**
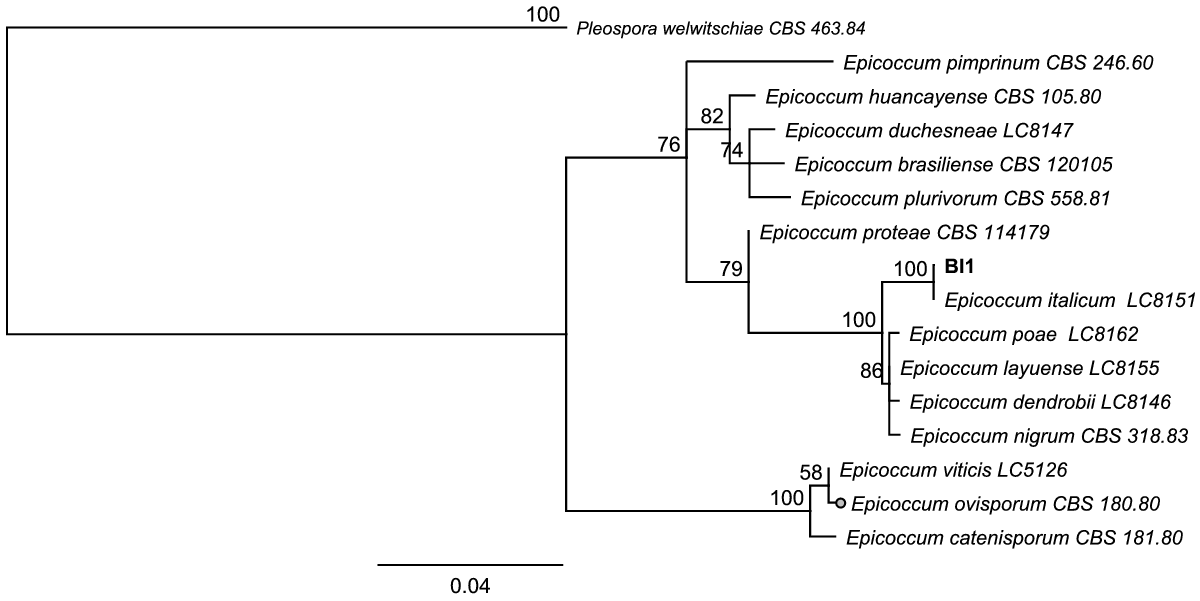
Neighbor-joining tree based on fungal internal transcribed spacer (ITS) sequences of Bl1 (Accession: OK090879), 14 *Epicoccum* species and *Pleospora welwitschiae* CBS 463.84 as outgroup. Numbers labeled at each node indicate bootstrap value (percentage) from 1000 replicates. Bar, 0.04 substitutions per nucleotide position.

**Figure 9:**
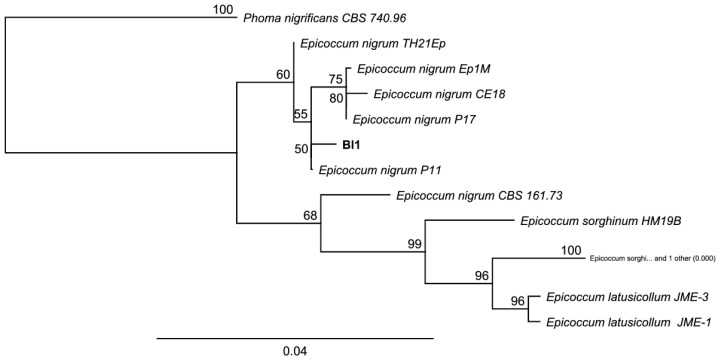
Neighbor-joining tree based on fungal tubulin sequences of Bl1 (Accession: OK319031), 11 Pleosporales species and *Phoma nigrificans* CBS 740.96 as outgroup. Numbers labeled at each node indicate bootstrap value (percentage) from 1000 replicates. Bar, 0.04 substitutions per nucleotide position.

**Figure 10:**
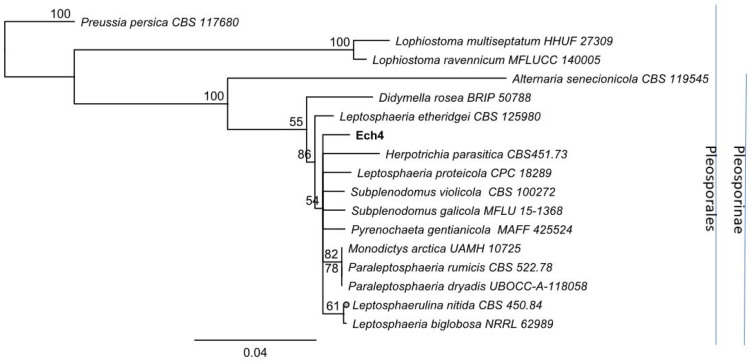
Neighbor-joining tree based on fungal internal transcribed spacer (ITS) sequences of Ech4 (Accession: OK090880), 15 Pleosporales species. and *Preussia persica* CBS 117,680 as outgroup. Numbers labeled at each node indicate bootstrap value (percentage) from 1000 replicates. Bar, 0.04 substitutions per nucleotide position.

**Figure 11:**
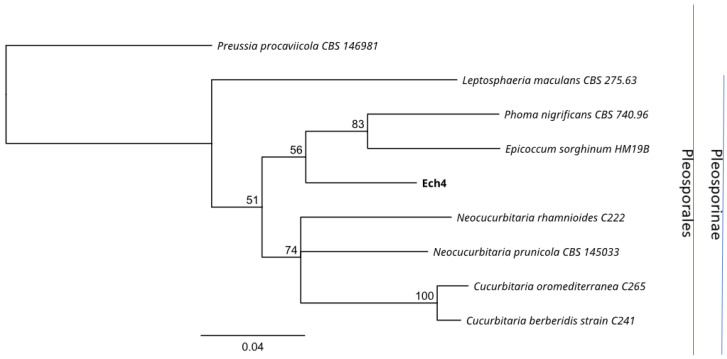
Neighbor-joining tree based on fungal tubulin sequences of Ech4 (Accession: OK319032), 8 Pleosporales species and *Preussia procaviicola* CBS 146,981 as outgroup. Numbers labeled at each node indicate bootstrap value (percentages) from 1000 replicates. Bar, 0.04 substitutions per nucleotide position.

For the molecular identification, the ITS region and β-tubulin marker were amplified for both Ech4 and Bl1 isolates. PCR products for the ITS sequence are shown as bands arounds 500 bp and 600 bp in the agarose gel and β-tubulin sequence shown as bands arounds 300 bp. Most similar ITS and β-tubulin sequences to the isolates were searched by an alignment using the NCBI BLASTn tool. The phylogenetic tree, based on ITS and β-tubulin sequences, showed isolate Bl1 and *Epicoccum* spp. are clustered in the same clade (Figure 8 and Figure 9). On the other hand, the phylogenetic tree based on ITS and β-tubulin showed that the isolate Ech4 is clustered in the same clade with more than one family, such as *Cucurbitariaceae*, *Leptosphaeriaceae*, and *Pleosporaceae* (Figure 10 and Figure 11).

## 4. Discussion

In this work, ten endophytic fungi were found in roots from plants growing in Chilean Central Precordillera. Six endophytes were found from the endemic plant *E. chiloensis*. Previously, an endophytic fungus belonging to the genus *Alternaria* was isolated from the mesenchymal tissue of this plant that inhabited in the same location [11]. This difference could be explained because the diversity of fungi occurring in shoots and roots may have important variations [37]. In addition, mycelial growth within plant tissues is heterogeneous [38], and the presence of endophytes and the synthesis of their metabolites may have marked seasonal variations [39,40]. Also, various isolates from the same fungal specie, obtained from separate plant sources, can produce different secondary metabolites [41].

On the other hand, in the endemic plant *B. linearis*, four endophytes were found, and this is the first report of endophytic fungi isolated from this plant.

Consequently, ten endophytes were isolated in this work, a similar number as other studies. For example, fourteen endophytic fungi were isolated from five endemic plants in India using a similar isolation method [42], and around five endophytes per plant from different gymnosperm plants were found in Chile [17]. On the other hand, also a higher number of endophytes have been reported in other studies, for example, a 108 endophytes were obtained from the endemic plant *Opuntia humifusa* in the United States [26], and 319 fungal species were isolated from the roots of 24 plant species from Spain [43]. Nevertheless, the relatively low number of endophytes found in this study could be explained by a rigorous disinfection of the plant surface or by different growing conditions, for instance, Silva-Hughes et al. incubated plant fragments for 60 days [26], an incubation period around four times longer than in this work. Out of ten endophytic fungi, two fungi designated as Bl1 and Ech4 showed antifungal activity against *B. cinerea* in dual confrontation assays; this inhibition was similar to an endophytic *Alternaria* sp. against the same isolate of *B. cinerea* using the same experimental conditions [11].Other authors previously reported endophytic fungi with antifungal activity against *B. cinerea* in Chile, such as endophytes found in the endemic tree *Embotrium coccineum* [18], endophytic fungi isolated from *Artemisia absinthium* [44], *Penicillium janczewskii* and *Microsphaeropsis olivacea*, endophytes isolated from Chilean native gymnosperms [17], and *Alternaria* sp. and *Aureobasidium* sp. fungi isolated from Chilean endemic and native plants [11]. This suggests that the fungi found could have potential as a biocontrol agent of this phytopathogen.

In addition, it was shown that both Bl1 and Ech4 isolates inhibited the *B. cinerea* mycelial growth by antibiosis, similar to other endophytic fungi; for example, fungi isolated from *Theobroma cacao* showed antibiosis against pathogens such as *Moniliophthora roreri* and *Phytophthora palmivora* [45], and endophytic fungi isolated from *Aloe vera* also inhibited by antibiosis the mycelial growth of *Fusarium oxysporum* [46]. The inhibition halo found in both isolates indicates that the antifungal activity of the obtained endophytic fungi is due to the secretion of diffusible secondary metabolites to the culture medium.

The identification of the Bl1 and Ech4 isolates by phylogenetic analysis based on the molecular markers ITS-rDNA and β-tubulin suggests that both endophytes are grouped with species of the order Pleosporales. The order of the Pleosporales is one of the largest of *Dothideomycetes* [47]. In this order, families such as *Didymellaceae*, *Didymosphaeriaceae*, *Pleosporaceae*, *Hypsostromataceae*, *Cucurbitariacea*, and others have been identified, and, within this classification, epiphytic, endophytic, and phytopathogenic fungal species have been found [48]. In detail, the sequences of the isolate Bl1 are more closely grouped with the genus *Epicoccum*, while the Ech4 isolate is closely grouped with more than one family within the suborder Pleosporineae [48].

On the other hand, in the case of isolate Bl1, this grouping is confirmed with the morphological analysis of the axenic culture, where the presence of sporodochia with orange-pigmented conidiomata are characteristic to species belonging to the genus *Epicoccum*, such as *E. italicum*, *E. layuense*, and *E. poae*, and the difference between these species is the conidium size [49,50]. *Epicoccum* species have been extensively reviewed as ubiquitous phytopathogens that can be used as biological control agents [51]. *Epicoccum* spp. have been found in different plant parts [50] and may be endophytes [52] and even saprophytes or pathogens [53,54].

Axenic culture of the isolate Ech4 showed small ovoid-shaped conidia and pigmented pilose pycnidia, which is described for different species of the Pleosporales order, such as the *Phoma* genus [49,55,56]. However, *Phoma* is considered a group of paraphyletic species that are continuously renamed and reassigned to different families of the Pleosporinae suborder, in addition to some genera whose family are still considered *incertae sedis* [47,48,57]. Additionally, Hou et al. suggested that the use of conventional molecular markers such as ITS and β-Tubulin present limitations that can be improved with the use of the rpb2 molecular marker to arrive at more reliable phylogenies [58]. Thus, in this study it is only established that isolate Ech4 corresponds to the Pleosporales order.

Many studies have been described that fungi of the order Pleosporales and the genus *Epicoccum* have been isolated as endophytes and their antifungal activity against different phytopathogenic fungi has been demonstrated [59,60,61,62]. This is the first time that *Epicoccum* sp. have been isolated from *E. chiloensis* and that an endophytic fungus of the order Pleosporales have been isolated from *B. linearis*.

On the other hand, in this work, the results suggest that both extracts obtained from the fungal isolates inhibit the mycelial growth of *B. cinerea*. Compounds with antimicrobial activity have been described for *Epicoccum* sp. and for many other Pleosporales species, such as *Coniothyrium* sp. and *Phoma* sp. [13,63,64]. Bioautography assays results suggest that orange active fraction of the extract from the isolate Bl1 (*Epicoccum* sp.), belongs to a group of pigmented compounds such as polyketides, carotenoids, and flavonoids already described in the genus *Epicoccum* [65]. Preliminary characterization of active fractions of the extract of the *Epicoccum* sp. isolate presented positive reaction with Dragendorff’s stain and iron (III) chloride solution, suggesting the presence of alkaloids and phenolics compounds in the extract. Alkaloids such as epicorazine A and epicorazine B have been described for this fungus and they have shown antimicrobial activity [66]. Also, aromatic polyketides with antifungal properties have been previously found in the *Epicoccum* spp. [67]. Meanwhile, active fraction of the extract from the isolate Ech4 suggest the presence of antifungal compounds. Some Pleosporales species of endophytic fungi such as *Phoma* sp. [59] and *Alternaria* sp. [11] produce metabolites with antifungal activity against *B. cinerea* [11,68]. Since the extract from Ech4 only showed a positive reaction for the vanillin/sulfuric acid stain, this suggests the presence of terpenes and their derivatives, such as saponins [69,70]. It has been reported that similar compounds have been described for fungal species of the order Pleosporales, such as *Leptosphaeria* sp. producing triterpenoid saponin to improve damage in their host, *Dipsacus asperoides* [71], and *Phoma* sp. producing terpenes, such as aphidicolin, a specific inhibitor of DNA polymerase, as well as antifungal compounds such as furan and dihydrofuran derivatives [64,72].

In conclusion, two endophytic fungi, *Epicoccum* sp. and *Pleosporales* sp., with antifungal activity against *B. cinerea* were isolated from the roots of plants growing in the Chilean Central Precordillera. This is the first report of *Epicoccum* sp. being isolated from *E. chiloensis* and of an endophytic fungus of the order Pleosporales being isolated from *B. linearis*. Also, possibly alkaloids and phenolic compounds were found in the extract from *Epicoccum* sp., and terpenes and their derivate compounds such as saponins from the fungus *Pleosporales* sp., could be responsible for the antifungal activity against *B. cinerea*, further study of these compounds and their effects may be a new alternative for the biocontrol of phytopathogens.

## Figures and Tables

**Table 1:** Endophytes isolated from the recollected plants.

Plant.	Fungal Isolate
*E. chiloensis*	Ech1
	Ech2
	Ech3
	Ech4
	Ech5
	Ech6
*B. linearis*	Bl1
	Bl2
	Bl3
	Bl4

## Data Availability

The data presented in this study are available on request from the corresponding author.
